# Effects of astrocyte on neuronal outgrowth in a layered 3D structure

**DOI:** 10.1186/s12938-019-0694-6

**Published:** 2019-06-18

**Authors:** Ao Fang, Dichen Li, Zhiyan Hao, Ling Wang, Binglei Pan, Lin Gao, Xiaoli Qu, Jiankang He

**Affiliations:** 1 School of Mechanical Engineering, Xi’an Jiaotong University, Xi’an, 710054 Shaanxi China; 2 State Key Laboratory for Manufacturing System Engineering, School of Mechanical Engineering, Xi’an Jiaotong University, Xi’an, 710054 China

**Keywords:** Neurons, Astrocytes, Three-dimensional tissue culture, Brain-like tissue

## Abstract

**Background:**

Human brain models and pharmacological models of brain diseases are in high demand for drug screening because animal models have been found to be less than ideal for fully representing the human brain and are likely to fail during drug screening and testing; therefore, the construction of brain-like tissues is necessary. Due to the complexity of cortical tissue, the in vitro construction of brain-like tissue models has been restricted to mostly two-dimensional (2D) models and, on a limited scale, three-dimensional (3D) models.

**Methods:**

In this study, 3D tissue blocks encapsulating neurons and astrocytes were constructed and cultured in vitro to mimic the cortex of the brain and to investigate the effects of astrocytes on the growth of neurons in a 3D culture.

**Results:**

The results indicated that such methodology can provide a 3D culture environment suitable for neurons and astrocytes to live and function. When both cells were evenly mixed and cultured in a 3D manner, the astrocytes, which showed better outgrowth and a higher proliferation rate, benefited more than the neurons. On the other hand, the neurons benefited, showing longer axons and a denser network of dendrites, when they were accompanied by astrocytes at a certain distance.

**Conclusion:**

In conclusion, astrocytes stimulated the outgrowth of neurons in a 3D culture environment in vitro. Regardless, the spatial relationship between both types of cells should be controlled. Thus, culturing cells in a 3D manner is necessary to investigate the correlations between them. This study provides a foundation for biofabricating 3D neurons’ cultures to allow for a deeper insight into the relationship between astrocytes or other glial cells and neurons in a 3D culture that is similar to the natural environment of the brain.

## Background

The study of the human brain remains one of the most important pioneering branches, especially for investigations of brain diseases and pharmacological studies. However, the progress of these studies is impeded by the lack of efficient and feasible human brain models; non-mammalian and rodent models are unable to fully represent human brain diseases or evaluate therapies or drugs, whereas studies involving non-human primate models are generally too costly and slow [1, 2]. A feasible alternative to such a problem is to generate brain-like tissue with similar cell content and structure as that of the natural brain; brain-like tissues may not have the same function as that of brain but can at least provide a good model for studying the interconnected relationships between cells and the environment [3].

The methodologies of constructing brain-like tissue in vitro fall into two categories: those that use extracellular matrix (ECM) and those that do not. Tissue with structural support is constructed via encapsulating neurons or neural stem cells in matrix materials such as hydrogels and adding nutrients before being extruding the cells into multi-layer structures. For such a methodology, the structures are pre-defined via photoetching or 3D printing methods to guide neurite outgrowth in the engineered tissue [3–7]. Tissue generated by this type of methodology has been found to provide a better growth environment for neurons in comparison to that cultured on two-dimensional plates [8, 9], and basic neural electrophysiological characteristics are observed [3–8]. Another methodology of constructing brain-like tissue in vitro is the traditional tissue engineering methodology used to develop three-dimensional (3D) multi-layer neural tissue induced from human pluripotent stem cells. In this type of methodology, no pre-defined structures are required to guide the outgrowth of the neurons, and the 3D structure can be formed in a similar manner to that of development, and basic neural electrophysiological characters are also observed [10, 11]. However, the size of such engineered tissue is normally smaller than 4 mm due to nutrient restrictions and other environmental factors [11]. Overall, both types of methodologies can be feasibly used to construct 3D brain-like tissue with neurons exhibiting basic morphology and function; however, considering the size restriction of the tissue engineering method, engineered tissue with structural supports is more promising.

The neural cortex contains different types of neurons and glial cells; the latter is found to play important roles in the outgrowth of synapses, nervous system repair, and the metabolism of neurons [12–14]. Amongst all glial cells, astrocytes are the most common and are critical for the survival of neurons, especially during extreme hypoxia [15] or inflammation [16]. Astrocyte dysfunction has been found to be related to many neural diseases [17], possibly due to the neuroprotective effects of astrocytes in the presence of perfluorooctane sulphonate (PFOS) [18], ammonia [19], H_2_O_2_ [20], etc. Astrocytes can also provide trophic support to neurons, and some astroglial growth factors, including insulin-like growth factor (IGF), nerve growth factor (NGF), ciliary neurotrophic factor (CNTF), and basic fibroblast growth factor (bFGF), have been shown to exert protective or regenerative influences on neurons following traumatic, chemical, or ischaemic lesions in the brain [21]. It has also been reported that astrocytes can promote axonal growth during central nervous system (CNS) development [22] and improve the outgrowth of synapses [23]. Moreover, transplanted astrocytes stimulate axonal regeneration after spinal cord injury in adult rats [23, 24]. In addition, astrocytes can stimulate the formation of synapses, promote the density of synapses during the developing stage of neurons, and consequently modulate synaptic transmission and regulate neuronal excitability [13, 25–27].

In vitro studies have also been carried out to investigate the effect of astrocytes on neurons. The co-culture of astrocytes and neurons in the same wells but separated via a porous insert have shown that human astrocytes share the ability of murine astrocytes to strongly promote neuronal survival in vitro [13]. Furthermore, it has been demonstrated that reactive oxygen species levels are higher in a co-culture system than in a monoculture system of neurons alone, in which neurons and astrocytes are separately cultured on different areas of the same surface [14]. Overall, many studies carried out in the literature have proved interconnections between neurons and astrocytes in a two-dimensional (2D) culture system, whilst limited reports have found interconnections between these cell types in a 3D culture system, which is believed to be more representative of the in vivo environment [6, 28–33]. Therefore, it is of interest to determine the correlation between neurons and astrocytes when they are encapsulated together in a 3D environment in vitro.

In this study, 3D tissue blocks encapsulating neurons and astrocytes were constructed and cultured in vitro to mimic the brain cortex and to investigate the roles played by astrocytes in the growth of neurons in a 3D culture. Three groups were used in this study: neurons alone, neurons and astrocytes combined uniformly, and neurons and astrocytes encapsulated individually but stacked together in a layer-by-layer manner. The tissue blocks were cultured for 14 days, and morphological studies of both cell types, especially regarding the length and density of the axons and dendrites of the neurons over time, were carried out. In this way, the effects of astrocytes cultured at various distances on the growth of neurons were studied in a 3D manner.

## Methods

### Cell isolation

Primary cortical neurons were isolated from the cortices of 15-day-old mouse embryos (Kunming strain, FMMU, Xi’an). The cortical tissue was removed and immersed in HBSS (Hank’s balanced salt solution) (14175-095, Gibco, USA) at 4 °C. The tissue was cut into pieces and digested with 0.25% trypsin (0458, Amresco, USA) for 20 min at 37 °C and was transferred and digested further in DMEM (SH30022.01, HyClone, USA) containing 20% foetal bovine serum (FBS, 10270-106, Gibco). Finally, the medium was filtered through a 40-μm cell strainer (352340, Falcon, USA) to dissociate the cortical neurons. The isolated neurons were re-suspended in complete neurobasal media (21103049, Gibco) containing 2% B27 neural supplement (17504044, Gibco), 1% l-glutamine (G105425-25g, Aladdin, USA), and 1% penicillin/streptomycin (P1400-100, Solarbio, USA). After being counted, the cells were used for the subsequent experiment. Primary astrocytes were isolated from 1-day-old mouse pups using the isolation method as that used for the neurons, and the isolated astrocytes were then directly subcultured in DMEM (SH30022.01, HyClone) containing 20% FBS (10270-106, Gibco) and 1% penicillin/streptomycin (P1400-100, Solarbio) at 37 °C with 5% CO_2_. When the astrocytes reached the third passage, the cells were digested and counted for tissue construction.

### Cell encapsulation in type I collagen

A rat tail-derived type I collagen solution (4 mg/mL in 0.1 M glacial acetic acid) was mixed with complete neurobasal media at a ratio of 1:3. The pH value of the collagen and medium solution was adjusted to 7.4 before it was used to resuspend the cells at the specified concentration at 4 °C. The mixture of collagen and cells was moulded into a 10 mm × 10 mm × 2 mm block and kept at 37 °C for 40 min to cure. Then, the tissue blocks with embedded cells were cultured in complete neurobasal media at 37 °C with 5% CO_2_.

### The construction of brain-like tissue

Three types of brain-like tissue blocks embedded with neurons and astrocytes (each with a different cell concentration, as summarised in Table 1), which included the neuron alone tissue (the N group), the mixed neuron and astrocyte co-culture tissue (the N&A-M group), and the layered co-culture tissue (the N&A-L group), were constructed using the methodology described above. The ratio of astrocytes to neurons in the natural brain cortex is normally between 1:2 and 1:3 [34]. In this study, the volume of neurons was larger than that of astrocytes. Therefore, a larger ratio (1:2) was chosen for the N&A-L group. The same ratio of 1:2 was chosen for the N&A-M group as the control group.

**Table 1:** Summary of the content of the tissues biofabricated in this study

Group	Model illustration	Content
N group		Neuron alone tissue
		Neurons at a concentration of 4 × 10^6^ cells/mL
N&A-M group		Mixed neuron and astrocyte co-culture tissue
		Completely mixed neurons (4 × 10^6^ cells/mL) and astrocytes (2 × 10^6^ cells/mL)
N&A-L group		Layered co-culture tissue
		A 3 mm × 3 mm × 2 mm block of astrocytes (2 × 10^6^ cells/mL) surrounded by neurons (4 × 10^6^ cells/mL) to form a 10 mm × 10 mm × 2 mm block

**Fig. 1:**
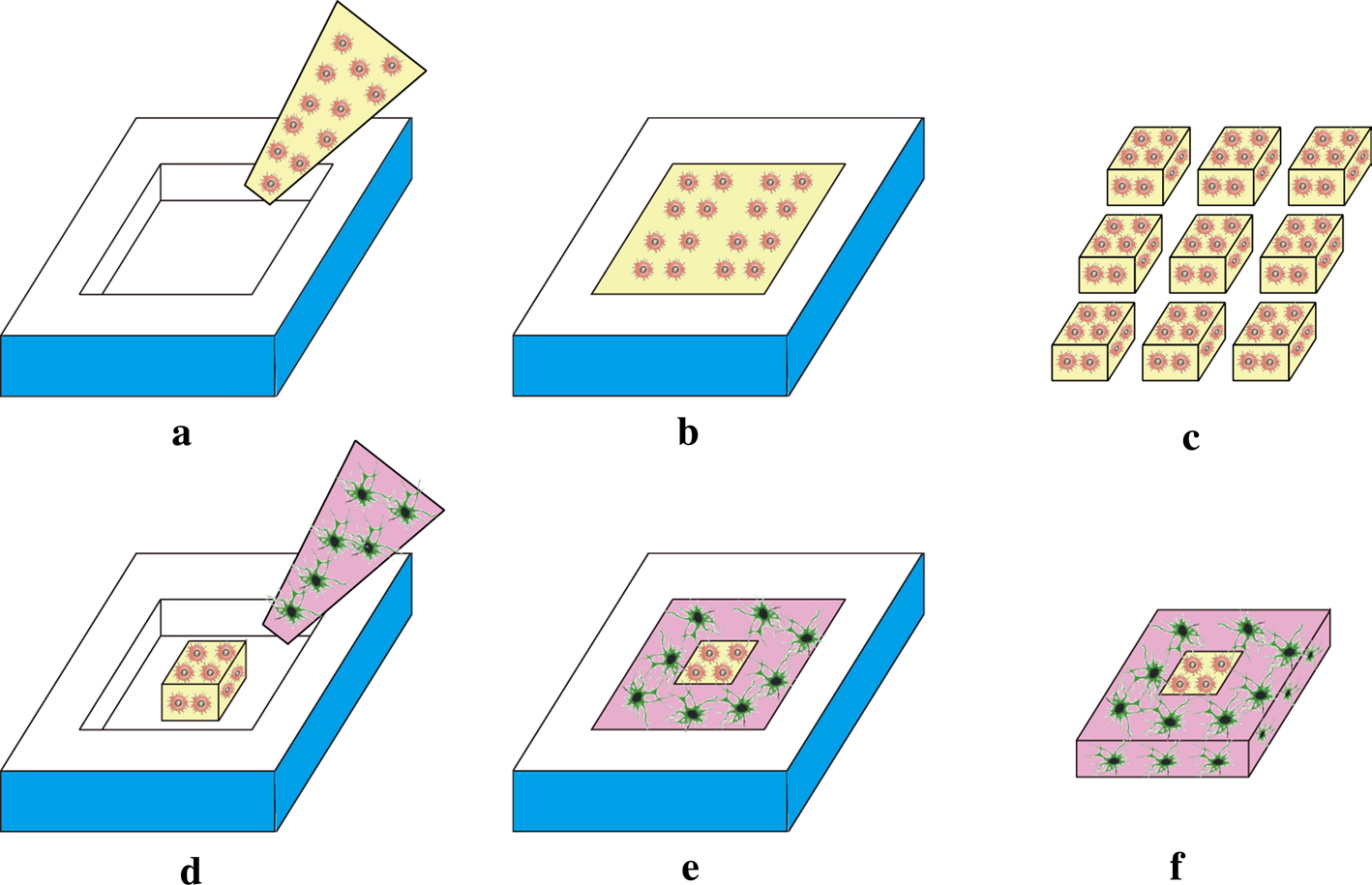
Diagram of the N&A-L model processing. **a** Mixing hydrogel with astrocytes; **b** forming the astrocyte mould; **c** cutting the astrocyte mould into small (3 mm × 3 mm × 2 mm) blocks; **d** surrounding the astrocyte block with hydrogel containing neurons; **e** forming the neuron–astrocyte mould; **f** the final N&A-L tissue

The N group tissue was embedded with neurons alone at a concentration of 4 × 10^6^ cells/mL, whilst the N&A-M group tissue was embedded with neurons and astrocytes at concentrations of 4 × 10^6^ cells/mL and 2 × 10^6^ cells/mL, respectively. The N&A-L group tissue was composed of two parts, one embedded with neurons alone at a concentration of 4 × 10^6^ cells/mL and one embedded with astrocytes alone at a concentration of 2 × 10^6^ cells/mL, constructed separately. The 3 mm (L) × 3 mm (W) × 2 mm (D) astrocyte part was moulded first, and then the neuron part was moulded surrounding the astrocyte part to make a combined 10 mm × 10 mm × 2 mm tissue block (Fig. 1). All tissue blocks were cultured in complete neurobasal media at 37 °C with 5% CO_2_. The media was changed every 2 days.

### Cell viability test

The cultured brain-like tissues were collected on different days. The LIVE/DEAD Viability/Cytotoxicity Kit (L3224, Thermo, USA) was utilised to study the cell viability of the brain-like tissues. A laser scanning confocal microscope (LSCM) (A1, Nikon, Japan) was used to image the stained cells. The living cells were stained with a green fluorescent marker and the dead cells with a red marker. The tissues were flattened, and the LSCM software NIS-Elements AR was used for cell counting. An average of 8 tissues were analysed at each timepoint. All the results were statistically analysed by one-way ANOVA.

### Immunofluorescence staining

The brain-like tissues were fixed with 4% paraformaldehyde for an hour and washed with 1× phosphate-buffered saline (PBS) 3 times at 5-min intervals. After being incubated in 1× PBS with 5% goat serum (AR0009, Boster, USA), a primary antibody diluted 1:200 and 0.3% Triton X-100 (T8200, Solarbio) overnight at 4 °C, the tissues were washed with 1× PBS 6 times at 10-min intervals and then incubated in 1× PBS with 5% goat serum, a secondary antibody diluted 1:200 and 0.3% Triton X-100 for 4 h before DAPI (AR1176, Boster) was added for an additional 10 min. Finally, the tissues were washed 6 times in 1× PBS at 10-min intervals and observed under an LSCM. All antibodies used are summarised in Table 2. The LSCM software NIS-elements AR was used for measuring and counting the length and the number of axons and dendrites.

**Table 2:** Summary of antibodies used in this study

Cell type	Primary antibody	Secondary antibody
Neurons	Rabbit anti-MAP2 (8707S, CST, USA)	Goat anti-rabbit IgG, Cy3-conjugated (CW0159S, CWBIO, China)
	Anti-neurofilament (2838S, CST)	Goat anti-mouse IgG, Cy3-conjugated (CW0145S, CWBIO)
Astrocytes	Mouse anti-GFAP (3670S, CST)	Goat anti-mouse IgG, FITC-conjugated (CW0113S, CWBIO)
	Rabbit anti-GFAP (16825-1-AP, Proteintech, USA)	Goat anti-rabbit IgG, FITC-conjugated (CW0114S, CWBIO)

### Electrophysiology

The electrophysiology of the neurons was measured by extracellular recordings. The electrode impedance was 4–6 MΩ, and the internal solution was 3 mol/L KCl. The position of the microelectrode was adjusted under a low-magnification objective lens. A positive voltage was applied to the electrode (immersed in ACSF containing 300 mmol KCl). The sample frequency was 10 kHz, and the frequency of the low-pass filter was 2 kHz. The recordings were analysed offline with pCLAMP 10.2 software.

## Results

### Survival rate of the neurons embedded in type I collagen

**Fig. 2:**
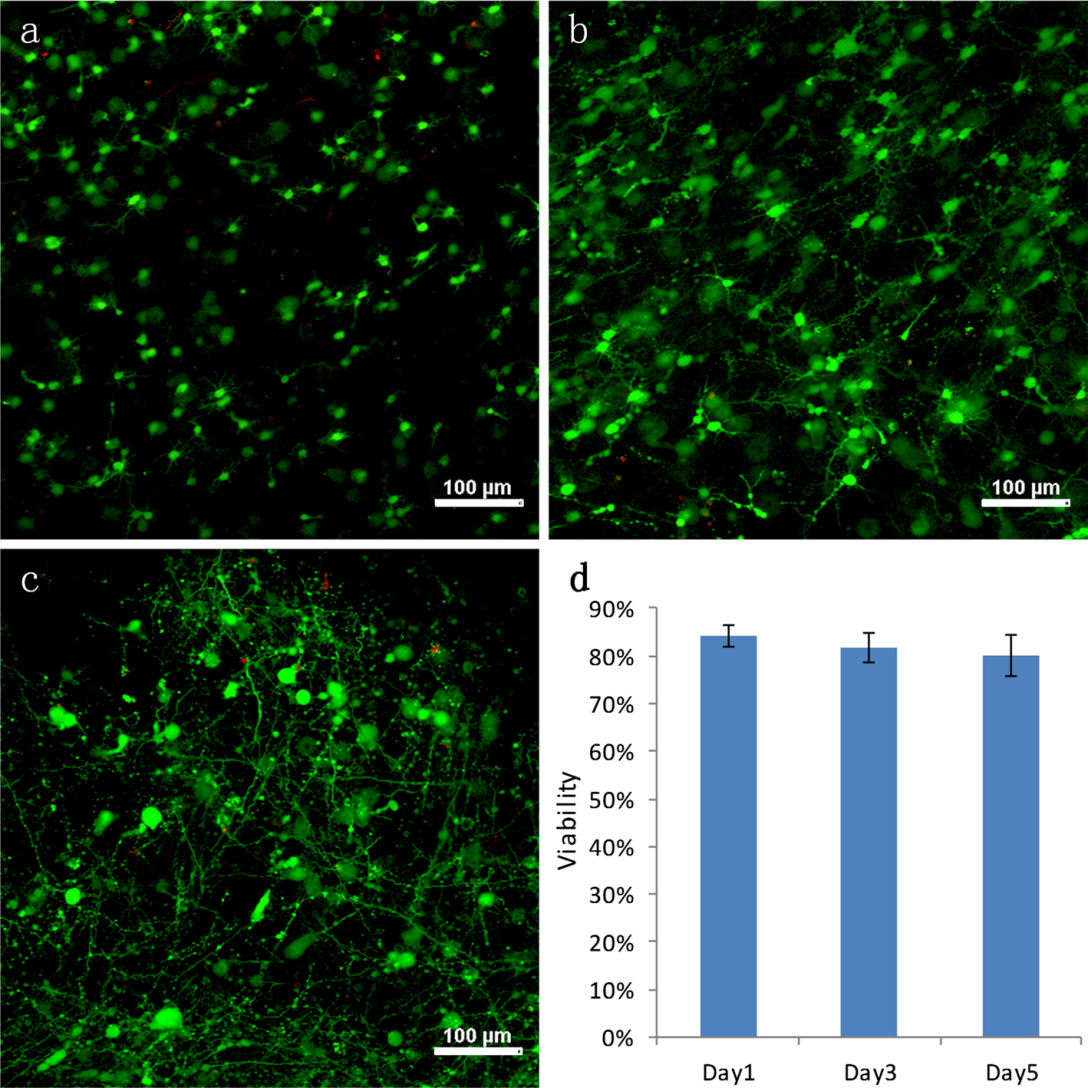
Fluorescence images of the neurons in the N group cultured tissue on day 1 (**a**), day 3 (**b**), and day 5 (**c**); **d** summarises the survival rate (*p* < 0.05)

The tissue blocks encapsulating neurons alone in type I collagen (the N group) were examined on days 1, 3, and 5 for cell morphology and viability (Fig. 2a–c: green represents the live cells and red represents the dead cells). Figure 2a–c indicates that the outgrowth of the neurons started on the same day as tissue construction, and the growth and interconnection improved rapidly with increasing culture time. The survival rate of the neurons was determined from the images and is summarised in Fig. 2d. The cell count results show that approximately 84 ± 2% of the neurons were alive on day 1, 82 ± 3% on day 3, and 80 ± 4% on day 5. Although there was a trend for the number of neurons to decrease, no statistically significant differences (*p* < 0.05) existed between the studied groups. This indicates that collage type I is an ideal biomaterial for in vitro 3D neurons cultures.

### Morphological studies of the cultured tissues

**Fig. 3:**
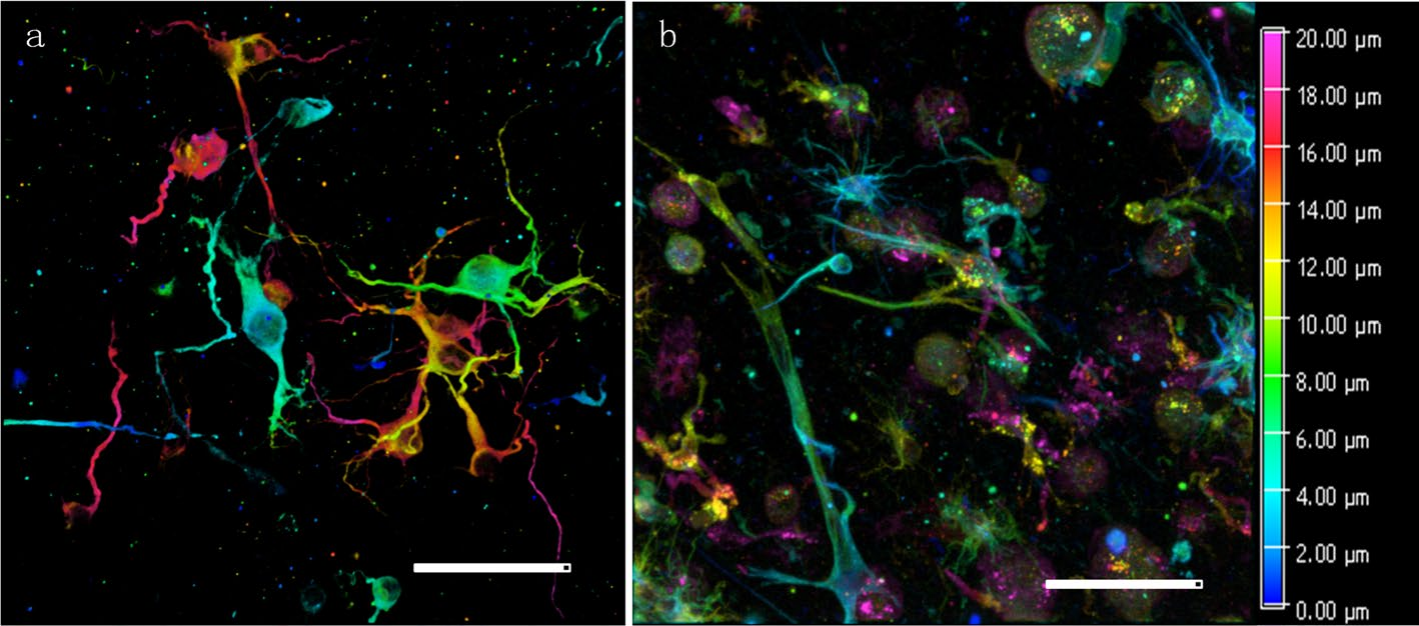
Fluorescence images of the neurons and astrocytes in N&A-L group tissue block on day 7. **a** Neurons (MAP2 positive) in 3D; **b** astrocytes (GFAP positive) in 3D. The colour bar denotes the depth along the *Z*-axis. Scale bar: 50 μm

The constructed N group (neuron), N&A-M group (mixed neuron/astrocyte), and N&A-L group (layered culture) tissue blocks were cultured for 14 days. Morphological studies were carried out on the tissue blocks of the three groups at various timepoints (days 1, 3, 7, and 14) via immunofluorescence staining and observation under an LSCM. Typical fluorescence images of the neurons and astrocytes of N&A-L group on day 7 are shown in Fig. 3. The images indicate that the neurons and astrocytes have spread well in the 3D culture environment.

### Morphological studies of the N&A-M group

**Fig. 4:**
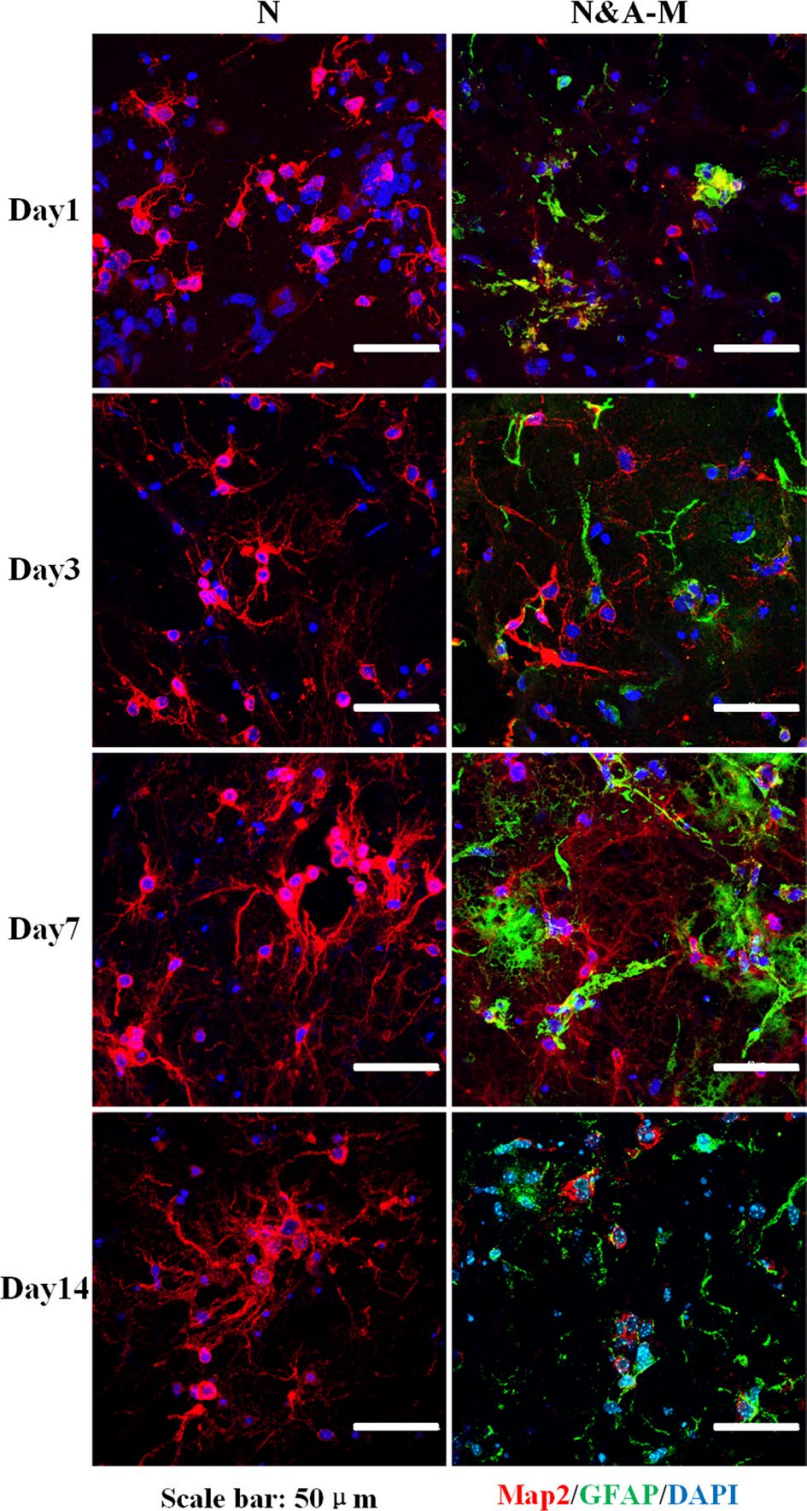
Fluorescence images of samples of the N&A-M group and the N group on days 1, 3, 7, and 14 (the neuronal dendrites were stained with MAP2 and are shown in red; the astrocytes were stained with GFAP and are shown in green; the cell nuclei were stained with DAPI and are shown in blue)

Morphological studies of the neurons of the N group and the N&A-M group were carried out via immunofluorescence staining (MAP2 staining of the neuronal dendrites is shown in red, GFAP staining of astrocytes is shown in green and DAPI staining of the cell nucleus is shown in blue), and the fluorescence images were observed under an LSCM. Typical fluorescence images of the neuronal dendrites and astrocytes at various culturing times are shown in Fig. 4. On day 1 of culture, the neurons in both groups showed similar morphologies; however, from day 4 to day 7, the neurons in the N&A-M group showed more greater dendrite growth than that in the N group. The number of neurons in the N&A-M group showed a large tendency to decrease on day 14.

### Morphological studies of the N&A-L group

**Fig. 5:**
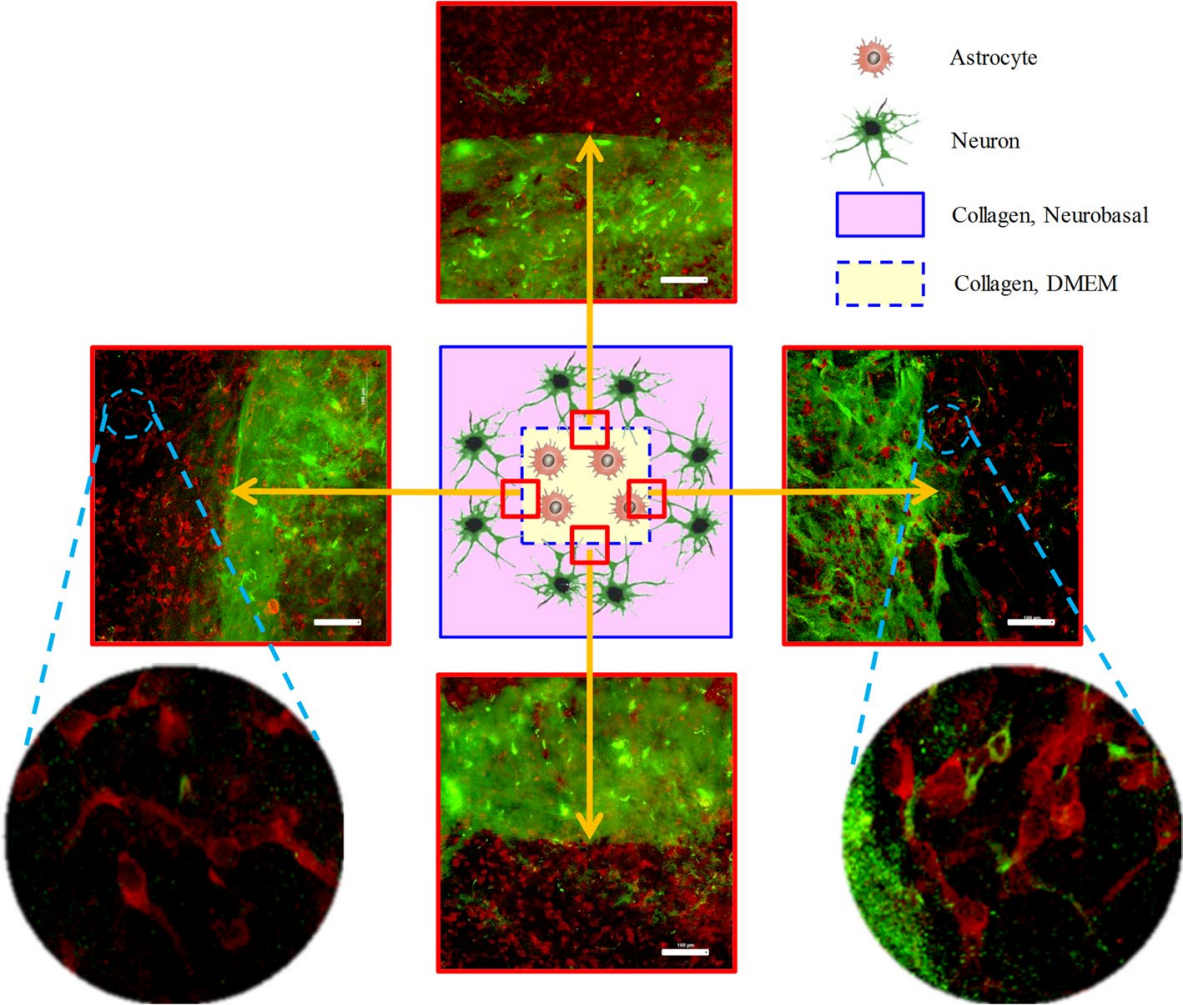
Images of the interface region between the neurons and astrocytes of the N&A-L group as observed under an LSCM (the astrocytes stained with GFAP are shown in green and the axons stained with neurofilament are shown in red). Scale bar: 100 μm

**Fig. 6:**
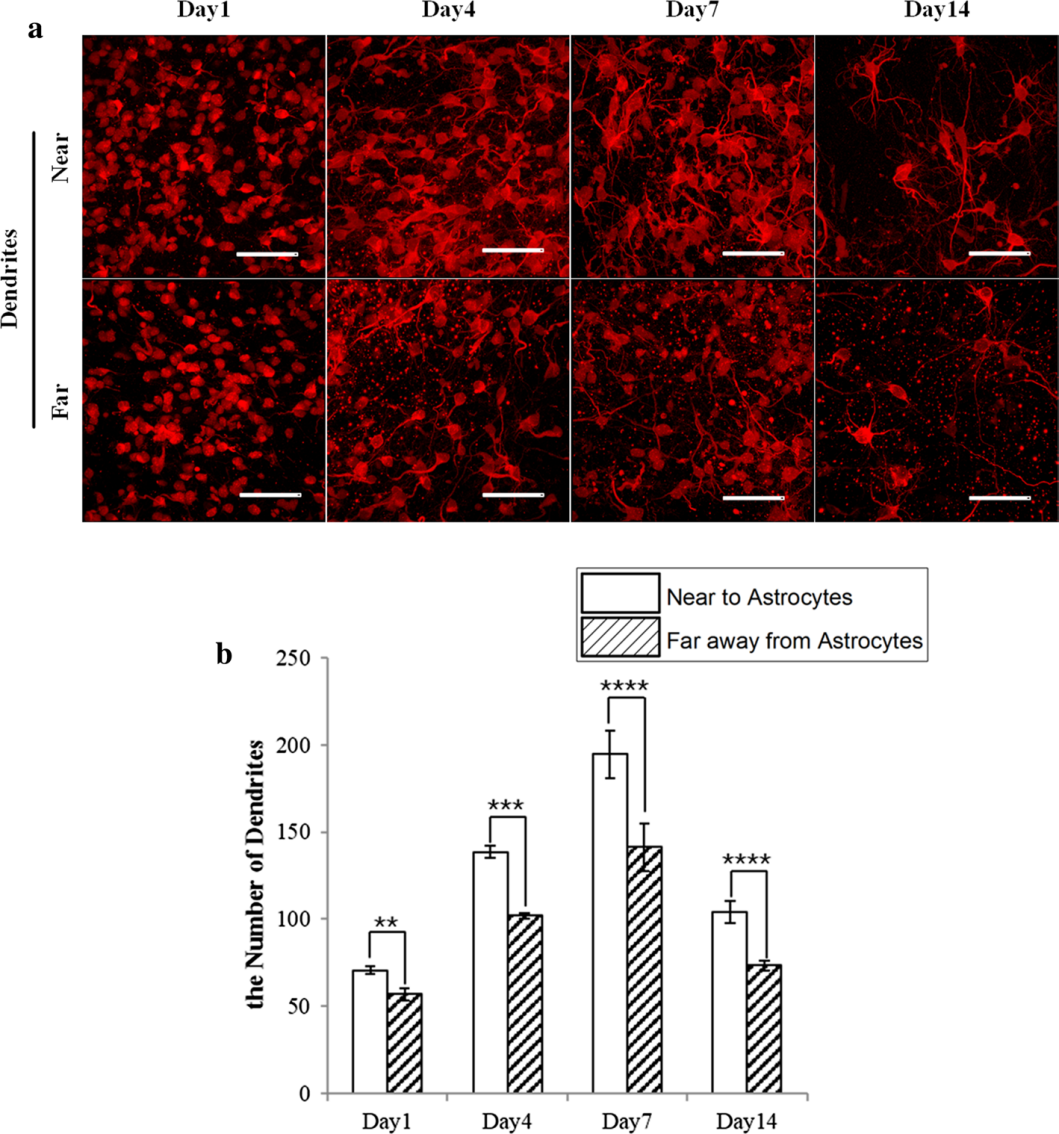
**a** Fluorescence images of the neuronal dendrites in the regions near and far away from the astrocytes at day 1, day 4, day 7, and day 14. Scale bar: 50 μm. **b** A comparison of the number of dendrites in the N and F groups in samples of the N&G-L group (*N* = 4) (*p < 0.05, **p < 0.01, ***p < 0.001, ****p < 0.0001)

**Fig. 7:**
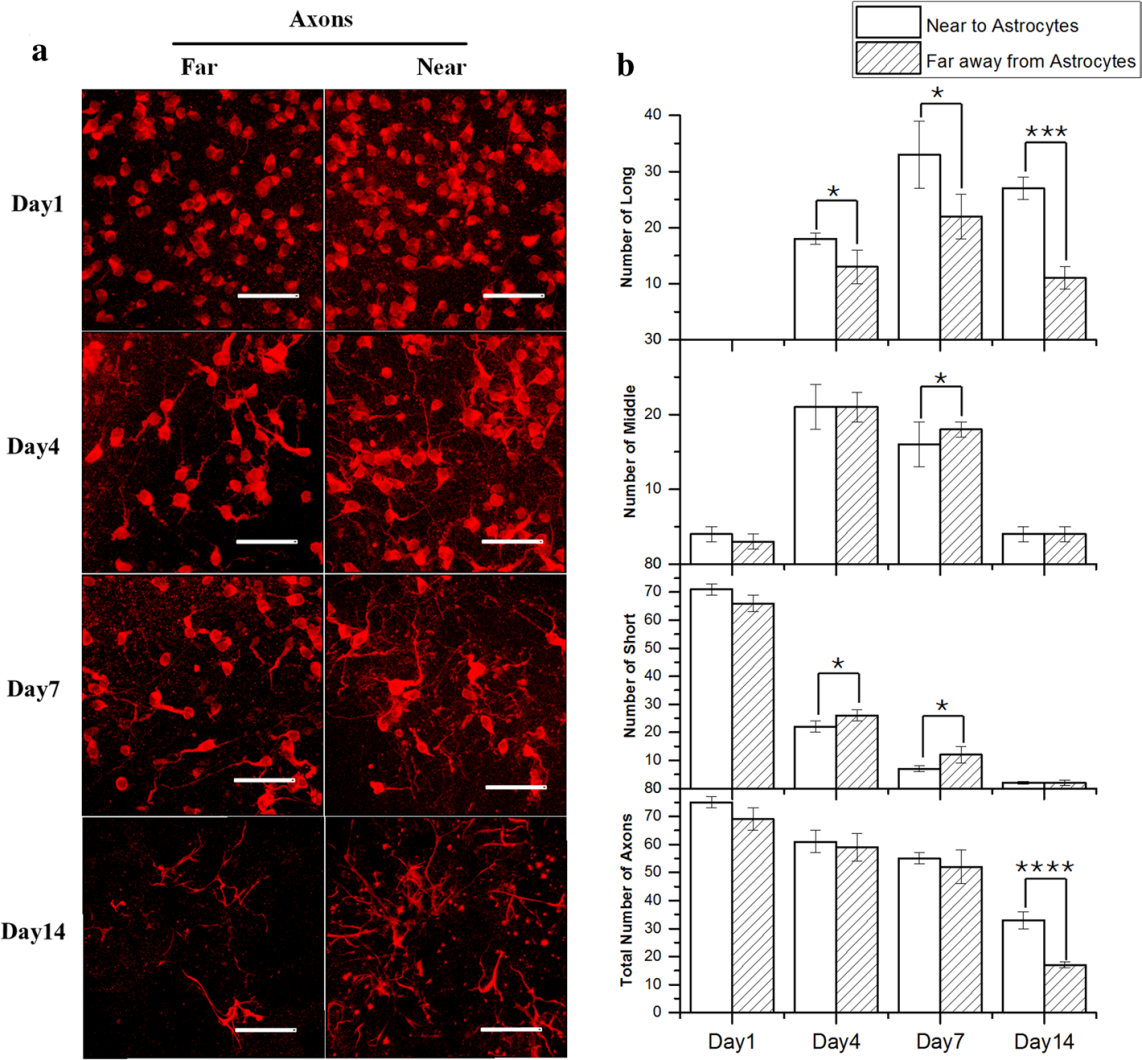
**a** Fluorescence images of the axons in the regions near and far away from the astrocytes on day 1, day 4, day 7, and day 14 (Scale bar: 50 μm); **b** A comparison of the number of short, long, and medium axons and the total number of axons in the N and F groups in the N&G-L group (*N* denotes the regions near the astrocytes; F denotes the regions far from the astrocytes, *n* = 4; Long: axons > 100 μm; middle: axon = 50–100 μm; short: axons = 0–50 μm (*p < 0.05, **p < 0.01, ***p < 0.001, ****p < 0.0001)

Morphological studies of the N&A-L group were carried out via immunofluorescence staining (MAP2 and neurofilament staining of neuronal dendrites and axons is shown in red and GFAP staining of astrocytes is shown in green), and the fluorescence images were observed under an LSCM. The interface between the neuronal region and the astrocytic region can be easily identified when observed under an LSCM in a 3D manner. The images of the typical interface at four locations of the tissue block are summarised in Fig. 5. The results indicate that the network of neuronal dendrites at the interface region was improved compared to that in the neuronal region (Fig. 6) and that relatively longer axons can be found passing through the interface region (Fig. 7).

### Effects of the spatial location of astrocytes on the functional growth of axons

Statistical analyses of the growth of axons at different timepoints were carried out. The following three categories of length were defined: short (0–50 μm), medium (50–100 μm), and long (> 100 μm). Four 208.51 × 208.51 μm^2^ zones on the four sides of the interface between the neurons and astrocytes, which are denoted as the region near the astrocytes (N), were selected. As a comparison, four 208.51 × 208.51 μm^2^ zones from the neuronal region, which were denoted as the region of far away from the astrocytes (F), were selected. For each selected zone, the axons and dendrites were counted and their lengths measured. In total, three samples were analysed, and the results are summarised in Fig. 6 (for the dendrites) and Fig. 7 (for the axons). The results indicate that, on day 1, most of the axons and dendrites in both groups were in the short category. On day 4, in the N group, the number of short axons decreased to 1/3 of the total number of axons, and medium and long axons each made up approximately 1/3 of the total number of axons. In the F group, the number of medium and long axons made up approximately 2/5 and 1/5 of the total number of axons, respectively. On day 7, in the N group, the number of long axons made up 1/2 of the total number of axons, and short axons only made up 1/8 of the total number of axons. In group F, long axons made up approximately 2/5 of the total number of axons, whilst the total number of axons in this group was lower than that in the N group. On day 14, in both groups, only long axons were present; however, the total number of axons in the F group was significantly lower than that in the N group. In general, the axons in the interface region of the N&A-L group were longer than those in the other region, indicating that the interface is a better growing environment for neurons.

### Effects of the spatial location of astrocytes on the functional growth of dendrites

Statistical analyses of the growth of dendrites in the regions near and far away from astrocytes were carried out by counting the total number of dendrites at different timepoints (Fig. 6) for samples of the N&A-L group. The results indicate that, on day 1, more dendrites were observed in the N group compared to the F group. Compared to day 1, on day 4, the total number of dendrites was increased dramatically in both groups, and more dendrites were observed in the N group compared to the F group. This increasing trend was maintained until day 14, at which point the number of dendrites in both groups decreased dramatically; however, more dendrites were found in the N group than in the F group. The results indicate that the interface is a better growing environment for neurons.

### Electrophysiology

**Fig. 8:**
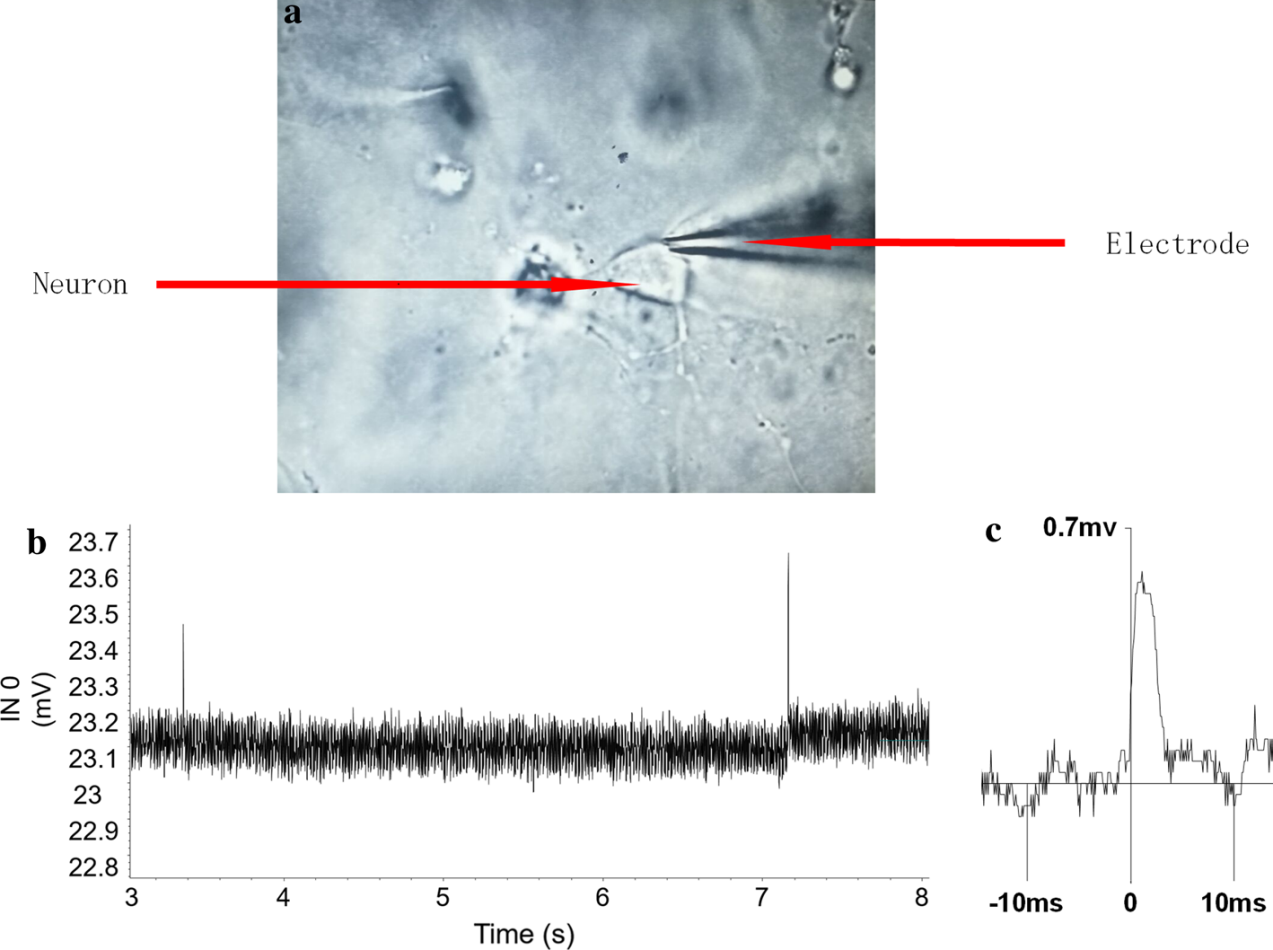
**a** The embedded neuron recorded by the electrode; **b** the diagram of potentials; **c** the extracellular spikes from the embedded neurons

The extracellular potential of the neurons embedded in the N group tissue block was recorded on day 7, as shown in Fig. 8a. The amplitudes of the field potential ranged from approximately 10 to 700 μV (Fig. 8b, c). The results show that the neurons embedded in collagen generated local field potentials. Thus, the neurons in the cultured 3D tissue block exhibit normal electrophysiological functions.

## Discussion

In this study, three-dimensional (3D) brain-like tissues were constructed in vitro by encapsulating neurons and astrocytes in hydrogel via moulding to study the role played by astrocytes in the outgrowth of neurons in natural brain tissue. To achieve this goal, three types of brain-like tissue models were constructed. One model involved the direct mixture of neurons and astrocytes (N&A-M), the second model combined individual blocks of neurons or astrocytes in a layered manner (N&A-L), and a monoculture of neurons alone (N) was also constructed as a control.

Analyses of the N&A-M group revealed a smaller number of healthy neurons, which indicates that astrocytic growth was overwhelmed compared to when both cell types were mixed together. This may be because of the unexpected overexpansion of astrocytes, which forms a dense network and occupies a large amount of space as well as nutrients within the tissue, therefore hindering the growth and function of neurons. Such a phenomenon has also been reported in two-dimensional (2D) co-culture scenarios in the literature [35] and may be caused by the better adaptability of astrocytes to culture environments compared to that of neurons. In this case, the 3D co-culture strategy was not more beneficial than 2D co-culture to the functional growth of neurons. Moreover, such a mixed model also brings great challenges to investigating the relationship between astrocytes and neurons and consequently restricts future application in the field of pathology and pharmacology.

Unlike the mixed model, the layered model (N&A-L) revealed a positive correlation between astrocytes and neurons starting on day 4, as indicated by the significantly denser network of axons in the zones close to the astrocytes compared to that in the zones far away from the astrocytes. The positive effect of astrocytes on the neurons was found to be enhanced when the tissue were cultured for 7 days, at which point long axons (> 100 μm) were the majority. The findings confirm the stimulating effect of astrocytes cultured with neurons in a 3D manner and agree with the findings of 2D culture scenarios reported in the literature [23]. According to the literature, immature astrocytes may help promote axonal regeneration and the growth of cortical axons and neurites. As such, an improved network is formed, and better communication between neurons is expected [23, 24, 36]. However, axons, which are responsible for conducting electrical impulses away from neuronal cell bodies to other neurons, are found to stretch out along the interface between astrocytes and neurons but are reluctant to enter the astrocytic zone. This phenomenon proves the preference of neurons for astrocytes; however, the astrocytic zone may not be a favourable environment for neurons, which agrees well with the findings obtained from the N&A-M group. On the other hand, astrocytes may be beneficial for stimulating and guiding the growth of axons.

A number of dendrites were found on day 1 of tissue construction (in the N&A-L group), as demonstrated by the staining results, and the number of dendrites increased dramatically as the time of culture increased. The number of dendrites peaked on day 7 for both the regions far away from and near the astrocytes. This indicates that the growth of neurons peaks on day 7 of 3D tissue culture, which is consistent with the results indicated by the growth of axons. Although the number of dendrites increased as the culture time increased, more dendrites were observed in the region close to the astrocytes than in the region far away from the astrocytes; the differences were significant (*p* < 0.01) beginning on day 1 and gradually increased until day 7. These findings reinforce the positive correlation between astrocytes and the formation of dendrites, which may involve the secretion of growth factors that benefits the outgrowth of neurons, which is consistent with the findings in the literature [13, 21, 25].

However, apoptosis was unavoidable in the neurons in both regions. The staining on day 14 demonstrated that the number of neurons as well as axons and dendrites was decreased sharply. This indicates that dendrites prefer the environment of the neighbouring region, which may be due to the chemical modification of the environment by the astrocytes, leading to a better nutrient status or the stimulation of signal transmission. This makes the layered model (N&A-L) a very good candidate for studying neuronal behaviour in vitro in a 3D manner and providing controlled stimulation from astrocytes similar to that in the in vivo environment. In addition, this provides another opportunity for drug screening and pharmacological modelling for the study of brain diseases.

Finally, field potential signals were obtained from the neurons cultured in the 3D tissue blocks on day 7, and the amplitude of the signal fell within the normal range (usually between 10 and 1000 μV) of living neurons reported in the literature [32]. This normal electrophysiological activity demonstrated the normal function of the neurons within the constructed 3D blocks, which helps to validate the biofabrication methodology of the 3D brain-like tissues in vitro.

## Conclusions

In this study, a biofabrication methodology was developed to successfully encapsulate neurons and astrocytes in 3D hydrogels to form 3D brain-like tissues in which neurons can live and function. To determine the conditions under which astrocytes contribute to the growth of neurons, two types of 3D tissue models were constructed with different spatial relationships between neurons and astrocytes. The results indicate that such a fabrication methodology can provide a 3D culture environment for neurons and astrocytes to live and function. The evenly mixed model of neurons and astrocytes benefits astrocytes more than it benefits neurons. On the contrary, the layered model in which neurons and astrocytes were neighbours across an interface was found to provide a better opportunity for neurons to grow, as indicated by the longer dendrites and denser network of axons. The conclusion that astrocytes can be beneficial and even critical to neuronal survival in vitro can be drawn; however, the spatial relationship between astrocytes and neurons should be controlled, and a 3D culture environment is necessary to investigate the correlation between both types of cells. This study provides a basic foundation for deeper insights into the relationship between astrocytes and neurons in a 3D culture environment and the investigation of future applications of drug screening and pharmacopathology models for brain diseases. Future studies should focus on determining the effect of various spatial relationships between neurons and astrocytes on the growth and function of neurons.

## Data Availability

The datasets used and/or analysed during the current study are available from the corresponding author on reasonable request.
