# Correction to: Effects of astrocyte on neuronal outgrowth in a layered 3D structure

**DOI:** 10.1186/s12938-019-0707-5

**Published:** 2019-08-08

**Authors:** Ao Fang, Dichen Li, Zhiyan Hao, Ling Wang, Binglei Pan, Lin Gao, Xiaoli Qu, Jiankang He

**Affiliations:** 1 School of Mechanical Engineering, Xi’an Jiaotong University, Xi’an, 710054 Shaanxi China; 2 State Key Laboratory for Manufacturing System Engineering, School of Mechanical Engineering, Xi’an Jiaotong University, Xi’an, 710054 China

## Correction to: BioMed Eng OnLine (2019) 18:74 10.1186/s12938-019-0694-6

It was highlighted that the original article [1] contained an error in the Acknowledgments section.

This Correction article shows the incorrect and correct Acknowledgments section.

**Incorrect statement**

The work was supported by the Program of the National Natural Science Foundation of China [51675411], and the Fundamental Research Funds for the Central Universities, GF Scientifc Innovation Scheme.

**Correct statement**

The work was supported by the Program of the National Natural Science Foundation of China [51675411].
